# SNPhylo: a pipeline to construct a phylogenetic tree from huge SNP data

**DOI:** 10.1186/1471-2164-15-162

**Published:** 2014-02-26

**Authors:** Tae-Ho Lee, Hui Guo, Xiyin Wang, Changsoo Kim, Andrew H Paterson

**Affiliations:** 1 Plant Genome Mapping Laboratory, University of Georgia, Athens, GA 30602, USA; 2 Department of Crop and Soil Sciences, University of Georgia, Athens, GA 30602, USA; 3 Department of Plant Biology, University of Georgia, Athens, GA 30602, USA; 4 Department of Genetics and Institute of Bioinformatics, University of Georgia, Athens, GA 30602, USA; 5 Institute of Bioinformatics, University of Georgia, Athens, GA 30602, USA; 6 Center for Genomics and Computational Biology, School of Life Sciences and School of Sciences, Hebei United University, Tangshan, Hebei 063009, China

**Keywords:** Polymorphisms, Linkage disequilibrium, Maximum likelihood

## Abstract

**Background:**

Phylogenetic trees are widely used for genetic and evolutionary studies in various organisms. Advanced sequencing technology has dramatically enriched data available for constructing phylogenetic trees based on single nucleotide polymorphisms (SNPs). However, massive SNP data makes it difficult to perform reliable analysis, and there has been no ready-to-use pipeline to generate phylogenetic trees from these data.

**Results:**

We developed a new pipeline, SNPhylo, to construct phylogenetic trees based on large SNP datasets. The pipeline may enable users to construct a phylogenetic tree from three representative SNP data file formats. In addition, in order to increase reliability of a tree, the pipeline has steps such as removing low quality data and considering linkage disequilibrium. A maximum likelihood method for the inference of phylogeny is also adopted in generation of a tree in our pipeline.

**Conclusions:**

Using SNPhylo, users can easily produce a reliable phylogenetic tree from a large SNP data file. Thus, this pipeline can help a researcher focus more on interpretation of the results of analysis of voluminous data sets, rather than manipulations necessary to accomplish the analysis.

## Background

Since the *Arabidopsis* genome was completed [1], advanced sequencing technology has facilitated the whole genome sequencing of many plants of commercial or experimental importance [2-4]. Reference genome sequences and high-throughput data analysis also provide the basis for resequencing whole genomes or transcriptomes to answer questions about variations between cultivars, populations, and taxa. In a variation study, the distribution of single nucleotide polymorphisms (SNPs) and/or short insertions and deletions (indels) is the prime concern.

A variety of studies have begun to utilize and illustrate how to deal with extensive SNP data [5-10]. Particularly, phylogenetic trees have been used in many evolutionary studies to depict evidence about evolutionary relationships between or within organisms, and to study the evolution and functional innovation of genes [6,7]. However, there has been no easy-to-use pipeline to determine phylogenetic trees with the huge number of variants obtained from sequencing projects. One typical method to determine trees has been: 1) calculating *p-distance* from all SNP data between two samples, 2) making the *p-distance* matrix for all samples, 3) constructing a neighbor-joining tree with the matrix by a program such as ‘neighbor’ in the PHYLIP package [11] and 4) drawing the phylogenetic tree image by a program such as MEGA4 [12]. However, there are at least three points to be methodologically improved: 1) there is no consideration of LD (Linkage Disequilibrium) blocks which can cause bias of variants, 2) statistical tests need be improved to evaluate the level of confidence, and 3) users are required to manipulate large data sets step-by-step to obtain a phylogenetic tree. The snpTree server [13] provided solutions for the second and third points. However, the target of this web server was bacterial genomes which are much smaller than eukaryotic genomes and seldom if ever have LD blocks.

We developed a pipeline, SNPhylo (Additional file 1), permitting users to construct a phylogenetic tree from a file containing SNP data in VCF (Variant Call Format), HapMap format or GDS (Genomic Data Structure) format [14]. Here we introduce the pipeline with three examples that show the applicability of the pipeline.

### Implementation

**Figure 1:**
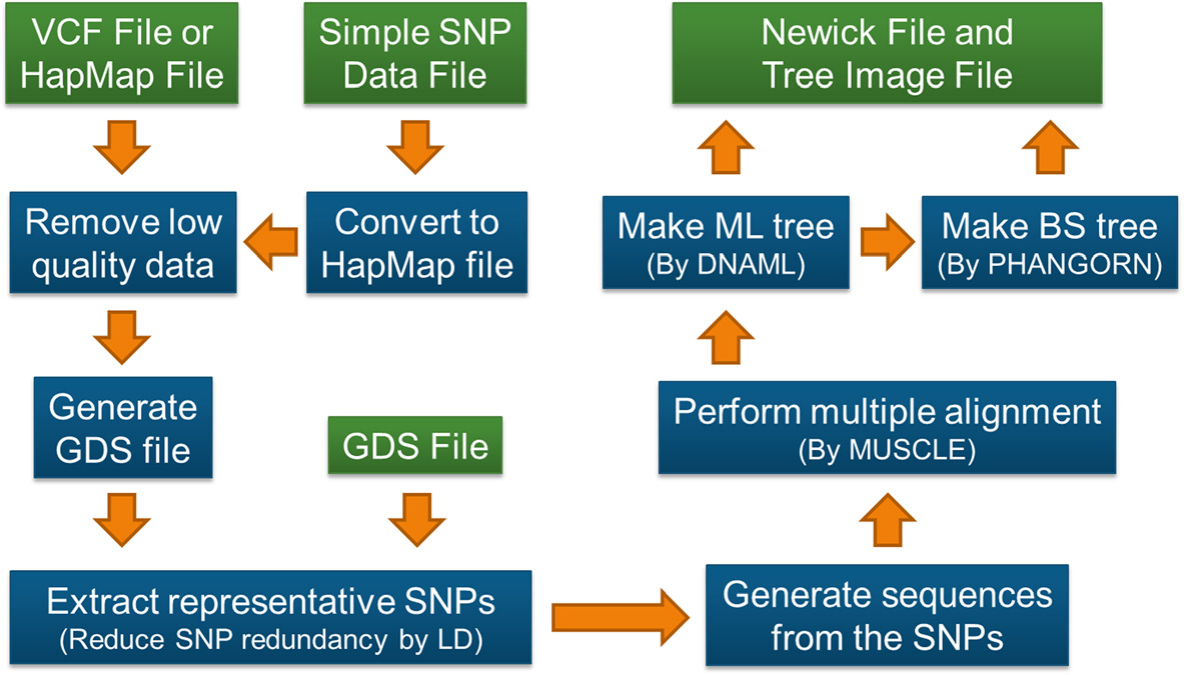
**Flowchart of SNPhylo pipeline.** The blue boxes represent processes in SNPhylo while green boxes indicate files as input and output. The orange arrows show the flow of data. By the SNPhylo pipeline, users can get a PNG format tree image file as well as a Newick format tree file determined from 4 different SNP data format file, VCF, HapMap, Simple and GDS.

Procedures to determine a phylogenetic tree in the pipeline are 1) testing each SNP position and removing those positions which do not have sufficient numbers of qualified SNPs for all samples, 2) generating new GDS format files from the tested SNP data files, 3) reading the GDS file and extracting SNP data which meet criteria of ≥ MAF (Minor Allele Frequency) and ≤ missing rate threshold, and are in approximate linkage equilibrium with each other as determined by SNPRelate package [14], 4) Concatenating the extracted SNPs for each sample and generating a sequence file containing the sequences, 5) Performing multiple alignment of the sequences by MUSCLE alignment program [15], and 6) Determining a phylogenetic tree by the maximum likelihood method by running DNAML programs in the PHYLIP package [11]. In addition, bootstrapping analysis for the tree is fulfilled by ‘phangorn’ package [16] (Figure 1). Using a GDS file as the SNP data file avoids the first and second steps.

All the steps are automated by one Bash shell script, snphylo.sh, though the pipeline includes additional components implemented in Python and R. Thus, by the script, users can obtain from a SNP data file a phylogenetic tree file and other informative files such as multiple alignment results file in PHYLIP format, which can be used for additional analysis such as a parallel bootstrap analysis by PhyML [17]. The pipeline also generates a tree image in PNG format with R packages [16,18] so the user easily interprets the results of analysis. In addition, the tree file in Newick format is provided as well so users can make more informative tree image by other programs such as MEGA4 [12] and Newick utility [19] depending on the demands of users.

## Results & discussion

### Phylogenetic tree with soybean SNP data

**Figure 2:**
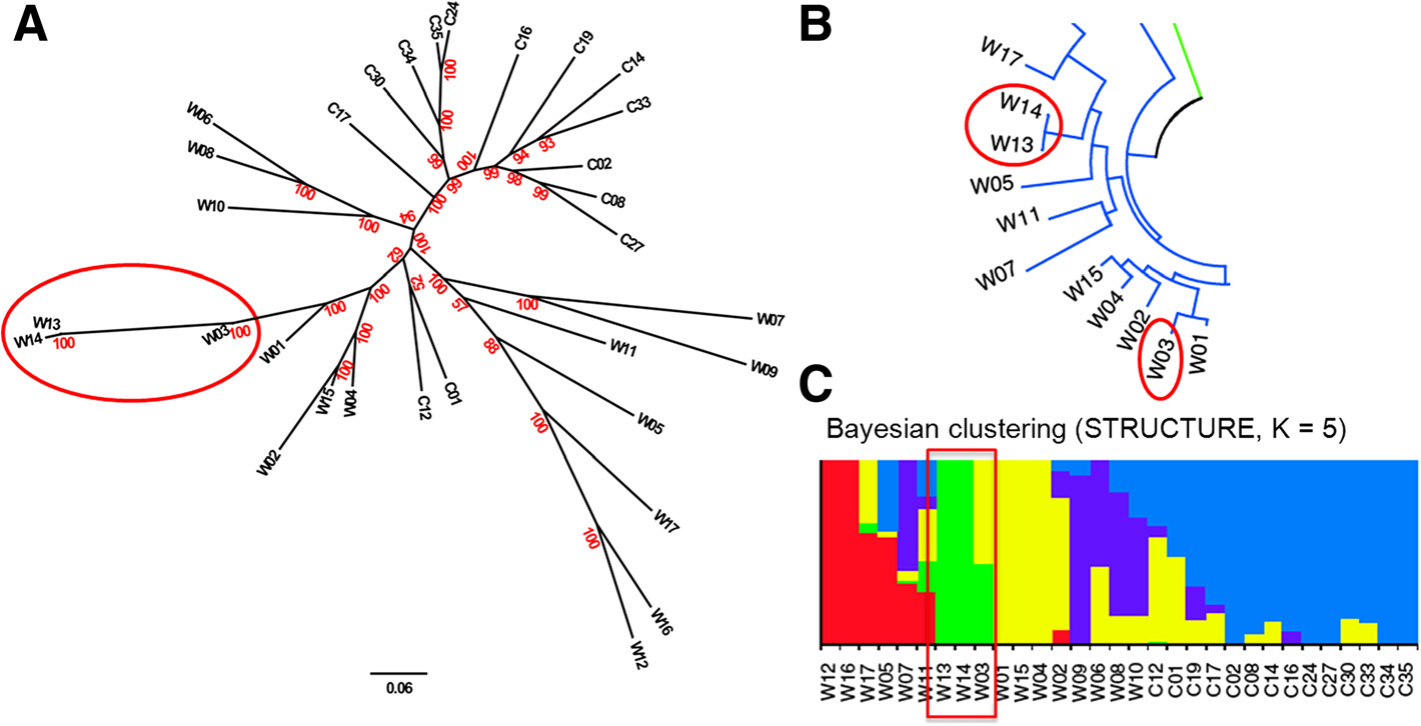
**Phylogenetic trees and Bayesian clustering result constructed with soybean SNP data. (A)** The tree constructed by SNPhylo pipeline with soybean SNP data from 31 soybean wild types and cultivars. The cluster which is more consistent with the Bayesian clustering result of the original report is circled in red. The ‘W’ and ‘C’ prefix in ID numbers represent wild type and cultivars, respectively. The bootstrap values determined with 1,000 samples are represented in red. **(B)** The part of tree of the original soybean SNP analysis report [7]. The IDs which are not consistent with the Bayesian clustering result are circled in red. **(C)** The Bayesian clustering result of the original paper [7].

As a demonstration of the use of SNPhylo, we determined a tree (Figure 2A) with published SNP data that includes 6,289,747 SNP loci determined by resequencing of 31 soybean wild types and cultivars [7]. The tree was determined with default options within 4 minutes using a GDS format file on a current Linux desktop computer which had 4GB memory and 2.66GHz Dual-Core CPU. In comparison, determination of the tree took about 50 minutes with a ~880 MB HapMap format file because of the need to perform additional steps that involve testing each SNP position and removing those positions which do not have sufficient numbers of qualified SNPs for all 31 samples, described in the procedures above.

Most branches in the tree correspond to those inferred in the original report [7] though our tree was easily determined by our pipeline in a relatively short time. Interestingly, in one case, our tree was more consistent with the Bayesian clustering result of the original report (Figure 2B) rather than the tree of the original report. Specifically, in the original report, the three wild soybeans (W03, W13, and W14) were clustered together in Bayesian clustering (red box in Figure 2C), while phylogenetic analysis separated W03 from the others (two red ellipses in Figure 2B). The tree determined by SNPhylo shows the three wild soybeans included in same cluster (red ellipse in Figure 2A), consistent with the Bayesian clustering result (red box in Figure 2C). In addition, we constructed a phylogenetic tree by the neighbor-joining method used in the original report using only the SNP data filtered by LD information, and obtained the same tree constructed by SNPhylo for the three wild soybeans (data not shown). Thus, the consistency with the Bayesian clustering result of both our tree and a phylogenetic tree based on LD-filtered data may indicate that using LD information improves interpretation of phylogenetic relationships from genomic data.

### Rapid construction of a tree with rice SNP data

**Figure 3:**
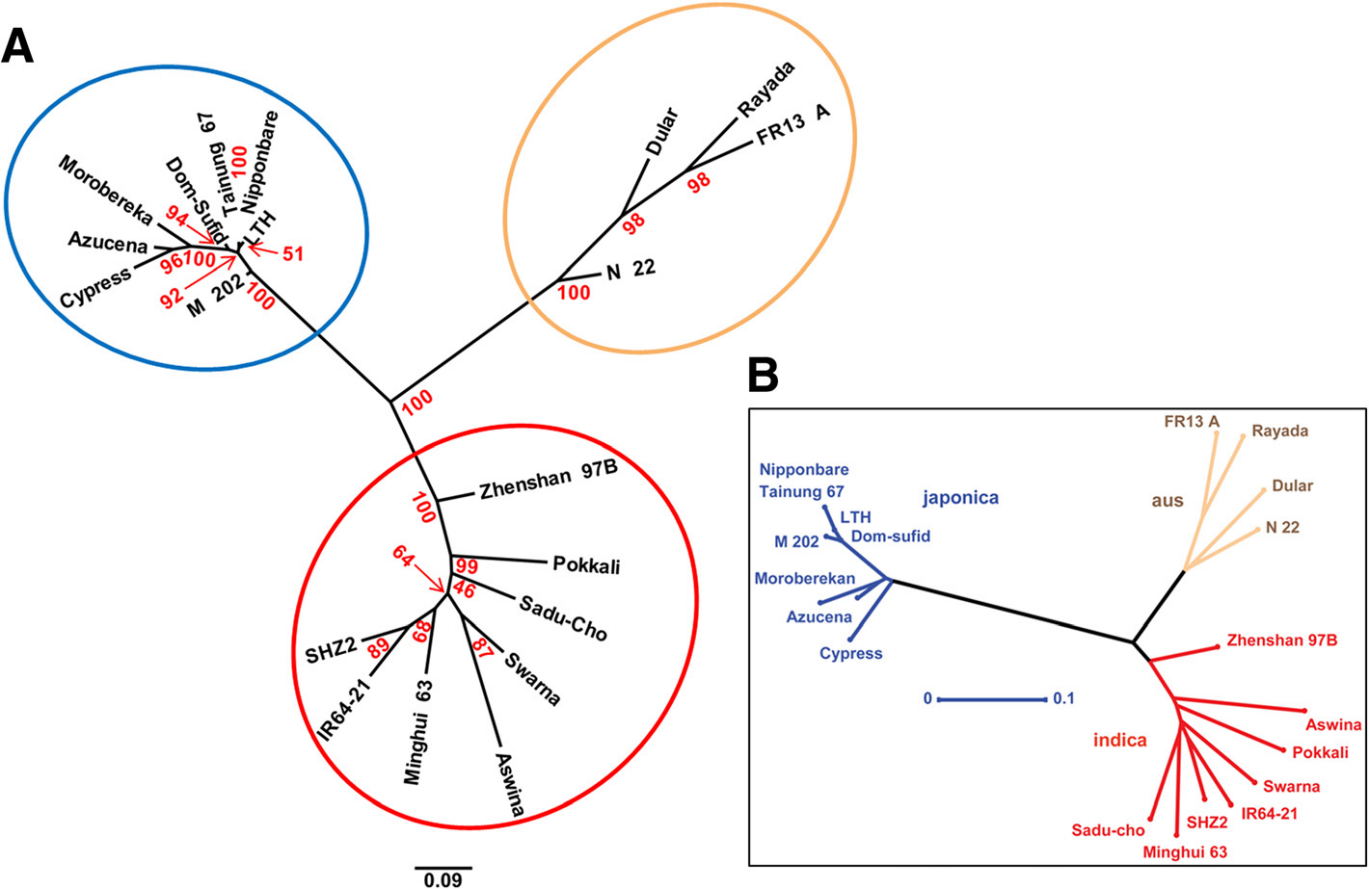
**Rice phylogenetic trees showing three rice groups. (A)** A rice SNP tree constructed by the SNPhylo. The three clusters in the tree reflect the three rice groups. The ellipses in red, blue and green represent japonica, indica and aus group, respectively. The bootstrap values determined with 1,000 samples are represented in red. **(B)** The tree in the original report for the rice SNPs [10]. The clustered in red, blue and green represents japonica, indica and aus group, respectively as well.

As another case study, we constructed a phylogenetic tree with rice SNP data that has 162,479 SNP loci determined by resequencing microarrays with 20 samples [10] (Figure 3A). Because of relatively low quality and small number of SNP data, the tree was constructed with loose parameters (−p 25) such that SNP loci were allowed to remain in the analysis even if as many as 25% of samples lacked data, versus the default of 5%. With the Linux system used to construct the soybean tree, the construction of the rice tree took less than 1 minute.

The tree constructed by SNPhylo had three evident clusters representing the three rice groups, japonica, indica and aus, and the results was consistent with the previous tree of the original report [10]. Interestingly, the previous tree (Figure 3B) and the SNPhylo tree showed different branch lengths between the three rice group clusters. Specifically, the branch between japonica and the other two clusters was much longer in the previous tree, with the branch lengths being more similar to one another in the SNPhylo tree. The relatively long edge in the previous tree may be caused by the higher LD level of japonica groups than other rice groups [10]. SNP bias due to high levels of LD in japonica might lead to overestimation of distances between clusters. The inclusion of a step to decrease this bias may permit SNPhylo to construct a more accurate tree.

### Construction of a phylogenetic tree with Arabidopsis SNP data

**Figure 4:**
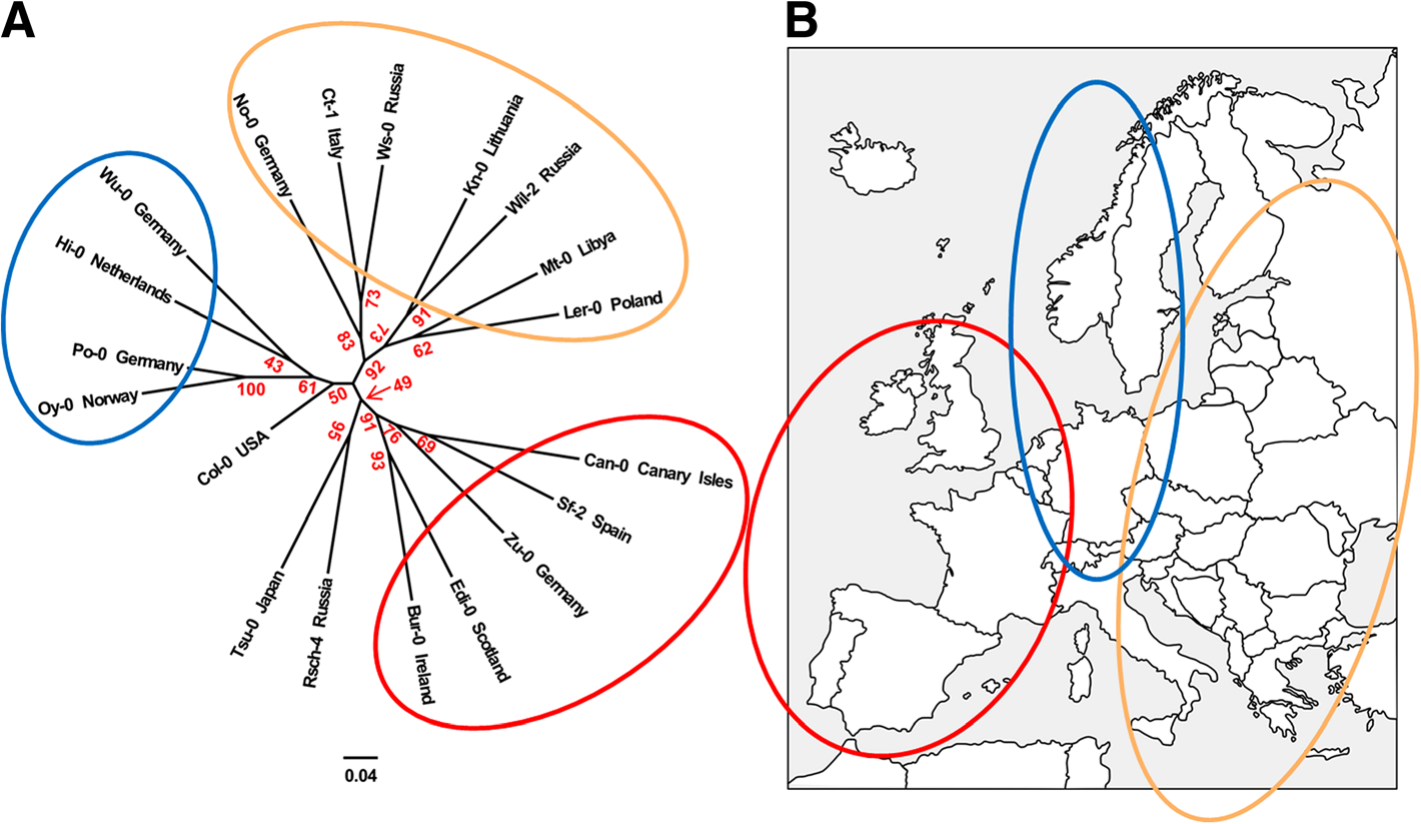
**Phylogenetic tree of *Arabidopsis *accessions and the geographical relations of the accessions. (A)** The *Arabidopsis* phylogenetic tree constructed with SNP data from 19 genomes by SNPhylo. Three major clusters in the tree are circled in three colors, red, blue, and orange. The bootstrap values determined with 1,000 samples are represented in red. **(B)** Europe continent map showing geographical relations of the accessions in each cluster in the Arabidopsis tree. The origins of accessions circled in red, blue, and orange are located in the geographical west, middle, and east of the continent, respectively.

*Arabidopsis* has been used as a model plant since its whole genome was sequenced [1] because of its small genome size, small physical size amenable to laboratory experiments, and short life-cycle. Since the first genome sequence was released, much *Arabidopsis* genome data has been released by various re-sequencing projects. Thus, as an additional case study, we constructed a phylogenetic tree with SNP data (Figure 4) determined by *Arabidopsis* genome project (http://mus.well.ox.ac.uk/19genomes/). Because of the relatively high LD level [20], the phylogenetic tree was constructed with relatively higher LD threshold (−l 0.4) than the default value.

There are three major clusters in the phylogenetic tree (Figure 4A). The accessions in each cluster show high consistency regarding geographic origins (Figure 4B). The origins of accessions circled in red, blue, and orange are located in the geographical west, middle, and east of Europe, respectively. For example, the origins of Edi-0 and Bur-0 in the same cluster are Scotland and Ireland, respectively. In addition, the relationship between geographical location and the cluster in the phylogenetic tree are consistent with the East–West gradient in clustering results of 96 *Arabidopsis* genotypes which is likely caused by post-glaciation colonization routes [21].

### Dependence of SNPhylo run time on amount of SNP data

**Figure 5:**
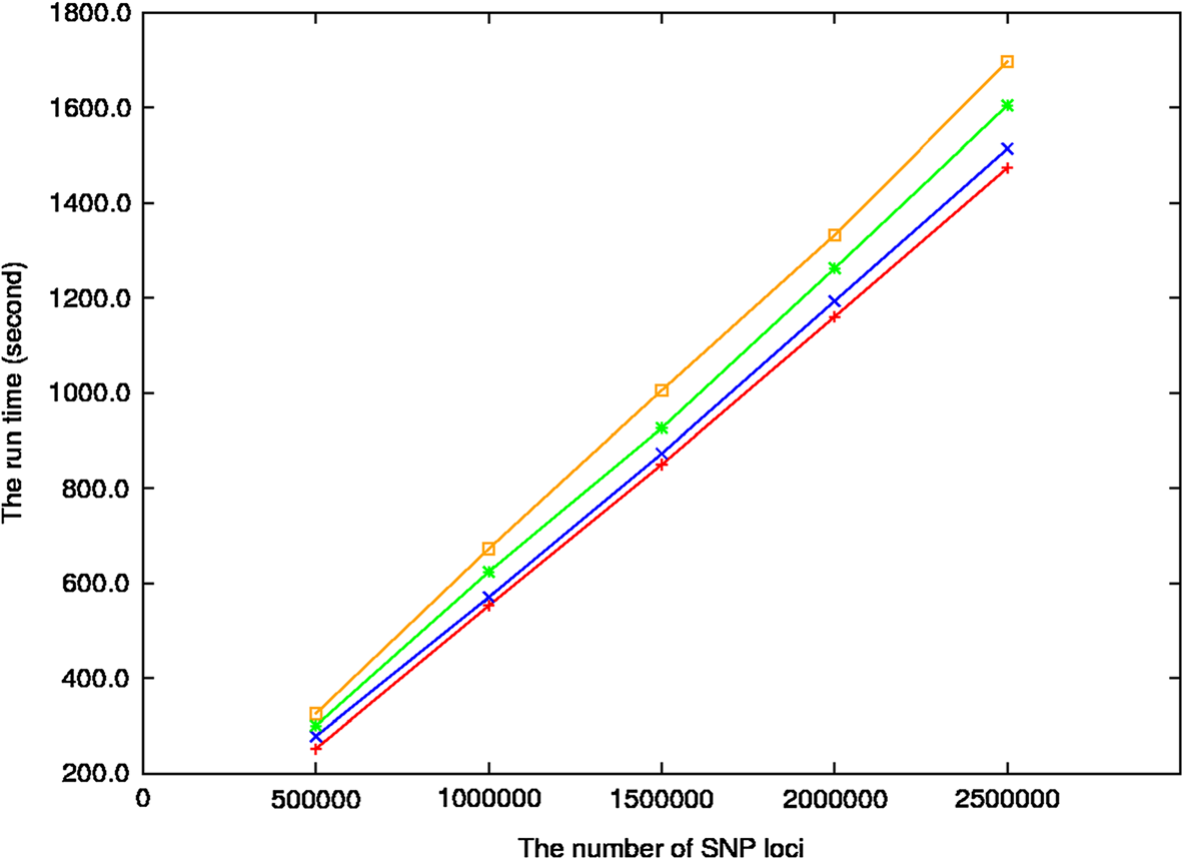
**Linear change of run time of SNPhylo depending the number of SNP loci.** The red, blue, green and orange lines represent changes of analysis time of HapMap files for 5, 10, 15, 19 Arabidopsis samples, respectively, depending on the changes of SNP loci number. Seeing the figure, the analysis time of SNP data is mostly affected by the SNP loci number rather than sample number.

The run time of the pipeline to generate a tree with the *Arabidopsis* SNP data for 2,595,179 SNP loci of 20 samples was 1,850 seconds. The result means that the pipeline can process about 1,402 SNP loci per second. However, it is not clear whether the number of SNP genotypes or the number of organism samples primarily determine the duration of the run. In order to address the question, we determined run times of the pipeline with various data sets generated from the *Arabidopsis* SNP data set in HapMap format (Figure 5). In the figure, each line shows the linear change of run time depending on the different number of SNP genotypes for a specific sample number. For example, the red line represents the nearly linear change of run time of SNP data sets of 5 samples by the number of SNP loci. The averages of run time for data sets having different SNP loci numbers for 5, 10, 15 and 20 samples are 857.4, 885.4, 943.1 and 1006.1 seconds, respectively. On the other hand, the averages of run times for data sets for 50,000, 100,000, 150,000, 200,000 and 250,000 SNP loci are 288.0, 604.7, 913.3, 1236.6 and 1571.8 seconds, respectively. The trends of the time changes in GDS format data (data not shown) were similar with the HapMap format data although the times were smaller than in the HapMap format. Therefore, the result shows that the run time of the pipeline is mostly affected by the SNP genotype number, rather than organism sample number.

## Conclusions

Using SNPhylo, users can easily produce a phylogenetic tree from large SNP data derived from various detection technologies such as genome wide resequencing [7] and resequencing microarrays [10]. Consequently, this pipeline can help a researcher focus more on interpretation of a reliable tree generated by maximum likelihood analysis of voluminous data sets, rather than manipulations necessary to accomplish the analysis.

### Availability and requirements

Project name: SNPhylo

Project home page: http://chibba.pgml.uga.edu/snphylo/

Operating system(s): Linux, UNIX and OS X

Programming language: Python, R and BASH

Other requirements: MUSCLE [15] and DNAML [11]

License: GNU GPLv2

Any restrictions to use by non-academics: None

## Abbreviations

LD: Linkage disequilibrium; VCF: Variant call format; GDS: Genomic data structure; MAF: Minor allele frequency.

## Competing interests

The authors declare that they have no competing interests.

## Authors’ contributions

THL developed the pipeline and wrote the manuscript. HG, XW and CK provided advice and revised the manuscript. AHP provided substantial advice and guidance during all phases of the project. All authors read and approved the final manuscript.

## Supplementary Material

Additional file 1 **SNPhylo version 20140116.** Description: This compressed file contains SNPhylo source codes and additional files such as setup script and instruction for installation. The latest version is available at SNPhylo homepage (http://chibba.pgml.uga.edu/snphylo/). Click here for file
